# Establishing evidence-based practices within services for children: knowledge transfer challenges

**DOI:** 10.1186/1472-6963-14-S2-P39

**Published:** 2014-07-07

**Authors:** Helene Eng, Camilla Lauritzen, Charlotte Reedtz, Willy-Tore Mørch, Monica Martinussen

**Affiliations:** 1 Regional Centre for Child and Youth Mental Health and Child Welfare, Northern Norway, UiT The Arctic University of Norway, 9037 Tromso, Norway

## Objectives

To facilitate implementation of evidence-based practice in the field of child and adolescent mental health in Norway.

## Background

Although more knowledge about effective interventions is frequently developed, most services in Norway are not evidence based. Part of the reason might be that practitioners and decision-makers do not know which interventions have scientific evidence for effectiveness and that they don’t search for information in traditional research literature. Although the evidence base of available interventions is growing, little research has been conducted on implementation strategies to bridge the gap between research and practice.

## Materials and methods

The Norwegian web-site http://www.ungsinn.no [http://ungsinn/] (Youngmind) has been developed to facilitate access to information about interventions and their evidence base. The website contains available interventions for the practice field in Norway. Each intervention is presented by a description followed by a classification of evidence level. A review of existing research with emphasis on effect studies is an important part of the presentation.

## Discussion

Establishing evidence-based practices within human services may be challenging, even though the practice in itself has been proven effective in efficacy and effectiveness research. To facilitate an evidence-based practice within psycho-social services for children, strategies should encompass more than just rigorous research on interventions. Knowledge transfer is a key concept, in terms of implementation quality and sustainability. A strategy that has a well-defined plan for knowledge transfer is more likely to be successful. It is important to simultaneously establish a substantial base of well documented interventions and additionally create strategies for successful knowledge transfer.

